# Organization of Astaxanthin within Oil Bodies of *Haematococcus pluvialis* Studied with Polarization-Dependent Harmonic Generation Microscopy

**DOI:** 10.1371/journal.pone.0107804

**Published:** 2014-09-12

**Authors:** Danielle Tokarz, Richard Cisek, Omar El-Ansari, George S. Espie, Ulrich Fekl, Virginijus Barzda

**Affiliations:** 1 Department of Chemical and Physical Sciences, University of Toronto Mississauga, Mississauga, Ontario, Canada; 2 Department of Chemistry, University of Toronto, Toronto, Ontario, Canada; 3 Department of Physics and Institute for Optical Sciences, University of Toronto, Toronto, Ontario, Canada; 4 Department of Biology, University of Toronto Mississauga, Mississauga, Ontario, Canada; 5 Department of Cell and Systems Biology, University of Toronto, Toronto, Ontario, Canada; University of Hyderabad, India

## Abstract

Nonlinear optical microscopy was used to image the localization of astaxanthin accumulation in the green alga, *Haematococcus pluvialis*. Polarization-in, polarization-out (PIPO) second harmonic generation (SHG) and third harmonic generation (THG) microscopy was applied to study the crystalline organization of astaxanthin molecules in light-stressed *H. pluvialis in vivo*. Since astaxanthin readily forms H- and J-aggregates in aqueous solutions, PIPO THG studies of astaxanthin aggregates contained in red aplanospores were compared to PIPO THG of *in vitro* self-assembled H- and J-aggregates of astaxanthin. The PIPO THG data clearly showed an isotropic organization of astaxanthin in red aplanospores of *H. pluvialis*. This is in contrast to the highly anisotropic organization of astaxanthin in synthetic H- and J-aggregates, which showed to be uniaxial. Since carotenoids *in vitro* preferentially form H- and J-aggregates, but *in vivo* form a randomly organized structure, this implies that astaxanthin undergoes a different way of packing in biological organisms, which is either due to the unique physical environment of the alga or is controlled enzymatically.

## Introduction

*Haematococcus pluvialis*, a freshwater species of Chlorophyta (a division of green algae), is known to synthesize and accumulate astaxanthin when faced with stressing environmental conditions including: high or low light intensity, high salinity, elevated temperatures and nutrient starvation [1]–[5]. Under one or all of these conditions, it is believed that astaxanthin is formed from β-carotene which is transported across the chloroplast membrane to the cytoplasm [6]. The increased accumulation of astaxanthin results in intense red coloration of the alga. The role of astaxanthin is believed to be the quenching singlet oxygen and reactive oxygen species generated during increased cellular stress, as well as a means to store energy and carbon for use when recovering from stressful conditions [7]–[10].

Astaxanthin has been reported to accumulate in oil bodies outside the chloroplast [6], [11], [12]. Fluorescence microscopy imaging of astaxanthin autofluorescence in stressed algae was found to coincide with fluorescence from oil bodies labeled with Nile Red [13]. Further determination of the location of astaxanthin in *H. pluvialis* has been performed by imaging the algae at varying growth stages with resonance-enhanced confocal Raman microscopy. Astaxanthin was found to be mostly concentrated towards the center of induced palmelloid cells and at the periphery of aplanospores [6]. Although studies have recently focused on the localization of astaxanthin within *H. pluvialis*, little research has focused on the organization and ultrastructure of astaxanthin within oil bodies. Astaxanthin is capable of forming H- and J-aggregates *in vitro* where an H-aggregate consists of closely packed astaxanthin molecules which are stacked with their conjugated chains oriented parallel to one another [14]. This is often referred to as a card-packed arrangement and it is characterized by a distinctive hypsochromic shift in the absorption spectrum of astaxanthin [14]. A J-aggregate consists of astaxanthin molecules arranged head-to-tail forming a more loosely packed aggregate differentiated from the H-aggregate by a bathorchromic shift in the absorption spectrum [14]. It has been hypothesized that astaxanthin molecules form H-aggregates in red aplanospores of *H. pluvialis* due to a characteristic shift of the C = C band in Raman spectra compared to the astaxanthin monomer [15].

In order to determine the localization and structure of astaxanthin accumulated within *H. pluvialis*, the alga was imaged with multicontrast nonlinear optical microscopy with three distinct contrast mechanisms including; multiphoton excitation fluorescence (MPF), second harmonic generation (SHG) and third harmonic generation (THG). In a nonlinear optical microscope, the signals are generated at high laser powers which is commonly achieved by utilizing a laser with 50–500 fs duration pulses for sample excitation coupled to a conventional confocal microscope. Optical sectioning is achieved without the requirement of a confocal pinhole since the nonlinear interaction only occurs at the focal spot due to the high energy requirement of nonlinear optical processes, which results in reduction in out-of-focus bleaching. MPF is similar to confocal fluorescence except the multiphoton excitation requires a combined energy of two or three infrared photons; for example, a 1028 nm laser induces two photon absorption at 514 nm. SHG and THG differs from fluorescence as they are determined by the nonlinear optical susceptibility of the molecules constituting the material as well as by the structural symmetry of the material. SHG and THG can be generated at wavelengths away from the absorption bands of the material and, therefore, have reduced photobleaching of the sample under those imaging conditions. In addition, generation of SHG and THG differs when detected in forward or backward directions with respect to the laser beam propagation, with stronger signals occurring in the forward direction [16], [17].

SHG is described by a second-order nonlinear optical susceptibility tensor, (*χ* ^(2)^), which is a function of the constituent molecules ability to produce SHG, as well as, their organization into a non-centrosymmetric structure. For example, intense SHG occurs in collagen [18]–[20], muscle [21], [22] and starch [23], [24], which have monomers (amino acids or glucans) that emit intense SHG, as well as inherent non-central symmetry in their ordering due to the helical arrangement of the polymers. THG is described by a third-order nonlinear optical susceptibility tensor, (*χ* ^(3)^), and relies on the ability of constituent molecules to produce THG, as well as their organization within the focal volume of the microscope. Unlike SHG, which requires the noncentral organization of molecules, THG requires structures that possess distinct interfaces within the focal volume. For example, chloroplasts [16], [17], [24], [25] emit intense THG due to the high third-order nonlinear optical properties of chlorophyll and carotenoid molecules [26], [27] in the light-harvesting proteins, as well as, their organization in stroma and grana, giving distinct interfaces. Similarly, crystalline beta-carotene aggregates [28] as well as cell walls [29] and membranes [30] provide structural conditions for THG. Therefore, nonlinear optical microscopy is a beneficial tool for non-invasive *in vivo* optical investigations of photosynthetic organisms such as *H. pluvialis*.

In order to determine the crystalline organization of astaxanthin in oil bodies, we employed the polarization-dependent harmonic generation method referred to as polarization-in, polarization-out (PIPO) harmonic generation microscopy [31]. The principle of a PIPO harmonic generation microscopy measurement is that the nonlinear optical susceptibility tensor values can be obtained via measurement of the orientation of the linear polarization of outgoing harmonic generation as a function of the linear polarization orientation of the laser radiation. Further, since the symmetry of a crystalline structure is reflected in the symmetry restrictions of the nonlinear optical susceptibility tensor, therefore, PIPO harmonic generation microscopy can be used to investigate the crystallographic structure of crystalline aggregates in microscopic samples. The most important feature of PIPO harmonic generation microscopy is that the structural organization of molecules can be determined in biological structures noninvasively and *in vivo*. For example, PIPO SHG has previously been used to discriminate between different collagenous tissues [31], [32], while PIPO THG has been used to obtain the molecular organization of β-carotene in orange carrots [28]. In this paper, comparison of PIPO THG data revealing the crystalline structure of astaxanthin within oil bodies of *H. pluvialis* and synthetic H-aggregates showed significant differences. Overall, this research implies that astaxanthin present in *H. pluvialis* undergoes a distinct method of packing which likely results from the physical environment within the alga or is controlled enzymatically. Studying the structural dynamics of crystalline carotenoid accumulation in different alga may aid the generation of knowledge to increase of the extraction yield of carotenoids for aquaculture, nutraceutical and pharmaceutical industries.

## Materials and Methods

### Theoretical Methods

**Figure 1:**
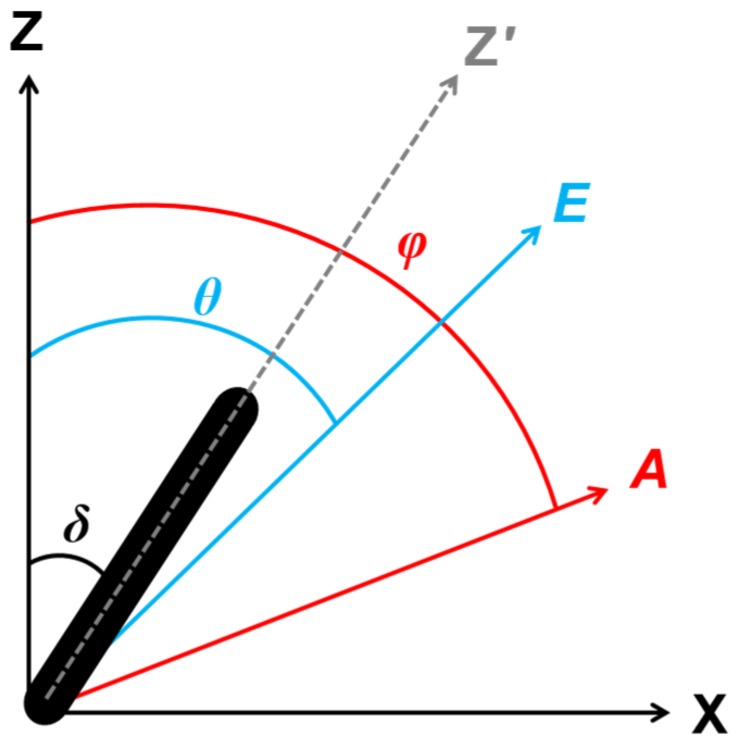
The orientations of coordinate frames in the experiment. The cylindrical axis (Z′) of a crystal (black cylinder) within a cell is oriented in the laser scanning plane (XZ) at an angle, *δ*, away from the laboratory Z-axis. The laser is directed perpendicularly (into the page) with the linear polarization oriented along *E*, at an angle, *θ*, from the Z-axis. The analyzer is oriented along *A*, at an angle, *φ*, from the Z-axis.

The second-order and third-order nonlinear optical susceptibility tensor component ratios were determined from PIPO SHG and PIPO THG measurements as follows [28], [31], [32]. The laboratory Cartesian coordinate system is defined with respect to the principal propagation direction of the scanning laser, XYZ, where XZ is the laser scanning plane, and the laser beam propagates along the Y direction (Fig. 1). A crystal is associated with another Cartesian coordinate system, with its cylindrical axis along Z′, which in the laboratory coordinate system is in the laser scanning plane, XZ, at an angle, *δ*, from the Z-axis (Fig. 1). If one dominant nonlinear dipole exists in the Z′ direction along the axis of the cylindrical structure, then two unique nonzero tensor components can be assumed, 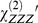 and 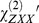 [28]. With this assumption, the general SHG intensity equation for PIPO measurements is as follows [32]:
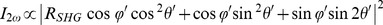
(1)where within the XZ laser scanning plane, *θ*′ = *θ-δ* is the angle between the incident polarization and the cylindrical Z′-axis, while *φ′* = *φ-δ* is the angle between the analyzer polarization orientation and the Z′-axis, and *R_SHG_* = 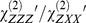. The prime term signifies that the susceptibility values and the polarizer and analyzer orientation angles are obtained for the cylindrical Z′-axis orientation. Note that when *δ* is equal to zero, the projection Z′-axis corresponds to the Z-axis of the XYZ laboratory coordinate system. Equation (1) shows the relation between the SHG intensity, *I*_2ω_ (commonly measured in photon counts) and the different analyzer and polarizer orientation angles as a function of the second-order nonlinear optical susceptibility ratio, *R_SHG_*.

Similarly, by assuming that a crystal with cylindrical axis along Z′ is in the laser scanning plane, XZ, and one dominant nonlinear dipole exists along Z′, then three unique nonzero tensor components can be assumed, 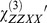, 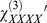 and 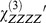 [28]. The general THG intensity equation for PIPO measurements is expressed as [28]:
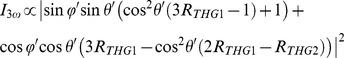
(2)where the prime term signifies that polarizer and analyzer angles as well as the third-order nonlinear optical susceptibility ratios are defined with respect to the projection Z′-axis, *R_THG1_* = 
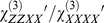
 and *R_THG2_* = 
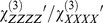
.

The numerical analysis of PIPO SHG and THG measurements yields the value of *δ*, the orientation of the crystalline axes in the XZ laser scanning frame, as well as *R_SHG_* (equation (1)), *R_THG1_* and *R_THG2_* (equation (2)). The values of *R_SHG_*, *R_THG1_* and *R_THG2_* can be analyzed to determine crystallographic structural information about samples. Furthermore, crystallographic variations between different regions in the sample can be measured down to the resolution of the laser focal volume in the XZ laser scanning frame, which in this experiment was ∼600 nm for SHG and ∼500 nm for THG [33].

### *Haematococcus pluvialis* cultivation

*Haematococcus pluvialis* UTEX 2505 was obtained from the Culture Collection of Algae at the University of Texas at Austin. The alga was cultivated on 1.5% v/v agar plates containing MES-volvox medium with a pH of 6.7. Cultures were incubated at 22°C. The cultures were continuously illuminated with cool-white fluorescent light at 30 µmol photons m^−2^·s^−1^. Light was measured with a light meter (LI-250A, LI-COR, Inc.) and a photometric sensor (LI-190SA, LI-COR, Inc.) in the 400–700 nm region of the electromagnetic spectrum. *H. pluvialis* cells were induced to accumulate astaxanthin by exposure to low light conditions of 5 µmol photons m^−2^·s^−1^ of cool-white fluorescent light.

*H. pluvialis* cells were lifted from the agar and quickly (30 s) immobilized in an 8% polyacrylamide gel [34]. The cells were immediately imaged with white light microscopy to check for uniform distributions of cells and then the cells were imaged with a nonlinear optical microscope in a temperature-controlled environment at 20°C.

### H-aggregate self-assembly of astaxanthin

A homogeneous methanol solution containing 50 µM astaxanthin (A 9335, Sigma-Aldrich Co.) was mixed with distilled water in a ratio of 1∶3 to induce formation of H-aggregates. The UV-Vis absorption spectrum of the mixture was recorded with an Olis-14 (upgraded Cary-14) spectrophotometer using a 1 cm Suprasil quartz cuvette (Hellma, Inc.) to determine which astaxanthin aggregate was formed. Formation of H-aggregates was confirmed by a large hypsochromic shift in the absorption spectra [14], [35].

### Nonlinear optical microscope setup

The laser source consisted of a femtosecond Yb:KGd(WO_4_)_2_ oscillator, which provided ∼450 fs duration pulses at a wavelength of 1028 nm with a pulse repetition rate of 14.3 MHz [36]. The laser was coupled into a home-built laser scanning microscope capable of MPF, SHG, THG detection, manipulation of the polarization of the laser light, and determination of the polarization of the emitted signals, as described in detail elsewhere [22], [31]. The scanning pixel dwell time was 2 ms. A high numerical aperture (NA) air objective (20×0.75 NA, Carl Zeiss Canada Ltd.) was used for imaging.

MPF, SHG and THG imaging was conducted simultaneously whereas PIPO SHG and PIPO THG measurements were performed sequentially. MPF was obtained with epi-detection. Three short wavepass filters (SPF-750, CVI Laser, LLC.) and one color glass filter (RG-665, CVI Laser, LLC.) was placed in front of the photomultiplier tube for separation of fluorescence from the laser radiation in MPF studies of *Haematococcus pluvialis*.

SHG and THG signal was collected in the forward direction through a home-built UV transmitting objective, then split with a dichroic mirror, and filtered with a band pass interference filter (F10-514.5, CVI Laser, LLC.) and a color glass filter, BG-39 (CVI Laser, LLC.) for SHG and F10-340 (CVI Laser, LLC.) for THG. The signals were measured using photon-counting detectors (MPF and SHG: H7421–40, Hamamatsu Photonics and THG: MP–1343, Perkin Elmer Inc.).

### Polarization-in, polarization-out (PIPO) measurements

A multicontrast nonlinear optical microscope was modified for PIPO measurements as previously described [31], [32]. Briefly, a half-wave plate (custom flat, Comar Optics Ltd.) was inserted before the excitation objective in order to rotate the linear polarization of laser light. The analyzer, a linear polarizer for the visible region (10LP-VIS-B, Newport Corp.), was placed after the collection objective for determination of the SHG polarization, while for THG polarization, a polarizer for the UV region (10LP-UV, Newport Corp.) was used.

SHG and THG images were collected at 132 sets of excitation and analyzer angles. The half-wave plate was rotated to 11 different polarization orientations evenly spaced between excitation angles from −90° to 90°, whereas the analyzer was rotated to 11 different evenly spaced polarization orientation angles from −90° to 90° for each excitation polarization angle. Reference frames to provide a baseline SHG and THG signal intensity were recorded every 11 measurements as an internal control.

For each 2×2 pixel area, the variation in SHG and THG intensity as a function of *θ* and *φ* was fitted using equation (1) for SHG and equation (2) for THG with a Levenberg-Marquardt algorithm. For fitting, *θ*′ and *φ*′ in equation (1) and equation (2) were replaced with 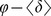 and 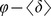 where 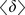 angle is between the laboratory Z-axis and the projection of the average cylindrical orientation axis of astaxanthin molecules in a pixel onto the XZ laser scanning plane (Z′-axis) [28], [31], [32].

Fitting of PIPO THG data was performed by fixing *R_THG1_* between −2 and 2 and allowing *R_THG2_* and 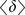 to be fit as free parameters. All code was written in MATLAB (MathWorks). Calibration of the initial orientation of the polarizer and analyzer with respect to the scanning frame in the microscope was performed using collagen from rat tail tendon [31].

## Results and Discussion

### Nonlinear optical microscopy images of *Haematococcus pluvialis*

**Figure 2:**
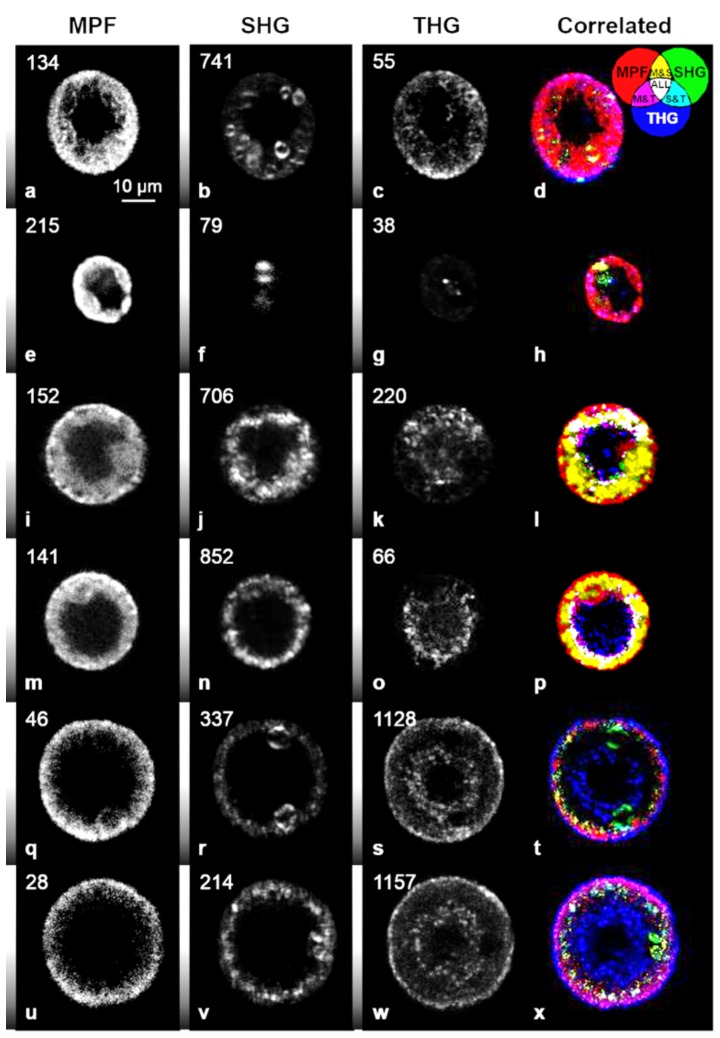
Nonlinear optical microscopy images of *Haematococcus pluvialis* at varying stages of light-stress. (a through h) palmella non-induced cells, (i through p) palmella induced cells and (q through x) aplanospores visualized by (a, e, i, m, q, u) MPF, (b, f, j, n, r, v) SHG, (c, g, k, o, s) THG and (d, h, l, p, t, x) structural image cross-correlation analysis between MPF, SHG and THG [16]. The numbers in the upper left corner indicate the maximum photon counts in a pixel and the grey scale bar on the left side of the image ranges from zero to the maximum signal intensity. In general, a large THG intensity difference is seen between green-colored palmella non-induced cells and red aplanospores where, for example, the red aplanospore (s) is about 20 times more intense than the green-colored palmella non-induced cell (c) due to increased concentration of astaxanthin.

Green palmella under non-inductive and inductive conditions, as well as red aplanospores were imaged with MPF, SHG and THG microscopy. Signals from the three contrast mechanisms highlight different features of the algae. Two-photon absorption of chlorophylls and carotenoids in the chloroplasts induces chlorophyll fluorescence [37], which is observed at the cell periphery (Fig. 2 a, e, i, m, q, u). During aplanospore development, the fluorescent region representing chloroplasts is reduced in size and intensity, as indicated by a reduction of the observed MPF signal per pixel, where the MPF signal from a stressed red aplanospore (Fig. 2q) is about 3 times less than the MPF signal of a green-colored palmella non-induced cell (Fig. 2a). This decrease in MPF signal intensity is likely due to the migration of carotenoids from the chloroplast during aplanospore development [6] as well as degradation of chloroplast components [13].

Second harmonic generation is observed from starch granules present in the algae at all stages (Fig. 2 b, f, j, n, r, v). Starch granules in the SHG images are characterized by their double-lobe structure [23], [24] near the cell periphery. The size of the large double-lobe structures seen in Fig. 2 b, f, r, and v was measured to be 5–7 µm and corresponds to the size of the starch capsule which surrounds the pyrenoid in the alga, in agreement with measurements by transmission electron microscopy (3–6 µm) [13]. Non-pyrenoid starch granules observed in TEM studies were typically ∼1 µm in diameter and are likely represented by the smaller SHG spots of similar intensity. In addition, the pyrenoids are observed in all stages of aplanospore development (Fig. 2 b, f, r, v). Thus, both induced and stressed cells retain the ability to fix CO_2_ as judged by the presence of pyrenoids [38] and retain the ability to produce starch through photosynthesis.

The THG signal intensity of the red aplanospore (Fig. 2s) is about 5 times higher than the signal of the green-colored palmella induced cell (Fig. 2k) and 20 times higher than the signal of the green-colored palmella non-induced cell (Fig. 2c). In green palmella under non-inductive conditions, weak THG signal is observed at the cell periphery (Fig. 2 c, g), which originates predominantly from the pigment-protein complexes in chloroplasts. In palmella under inductive conditions, more THG signal per pixel is observed (Fig. 2 k, o), however, the THG signal is largely present in the central region of the algae. In red aplanospores, more than an order of magnitude stronger THG signal is observed in both the central region of the algae and at the periphery (Fig. 2 s, w).

These observations are similar to what is seen in resonance-enhanced confocal Raman microscopy images of *H. pluvialis* which demonstrated the presence of chlorophyll, β-carotene and astaxanthin largely at the cell periphery in green-colored palmella non-induced cells, whereas in green-red colored palmella induced cells, chlorophyll remains at the cell periphery while β-carotene and astaxanthin are present in the middle of the cell [6]. In red aplanospores, chlorophyll and β-carotene are present at the cell periphery while astaxanthin is found at the periphery and in the middle of the cell [6].

In the green-colored palmella non-induced cells, correlation between all three nonlinear signals is not seen, however, there are regions where MPF and SHG or MPF and THG signals correlate. Correlation between MPF and SHG is seen where the SHG signal is generated due to starch granules present in the chloroplasts and MPF originates from the chlorophyll containing pigment-protein complexes in the chloroplasts (Fig. 2 d, h, l, p, t, x).

Correlation of MPF and THG signals is seen at all stages of cell growth. In particular, in the green-colored palmella non-induced cells (Fig. 2 d, h) and green-colored palmella induced cells (Fig. 2 l, p), the signals are correlated at the cell periphery which is likely due to the presence of both chlorophyll and carotenoid molecules in the chloroplasts. Both chlorophylls and carotenoids are known to have large third-order nonlinear optical properties. However, at 1028 nm wavelength laser radiation, the chlorophylls and carotenoids have opposite signs in their second hyperpolarizability values and therefore, partial cancellation of THG signal occurs [27], [37].

In red aplanospores (Fig. 2 t, x), correlated MPF and THG signals also occur at the periphery. The MPF signal is attributed to chlorophyll fluorescence while the THG signal is attributed to the aggregation of astaxanthin as astaxanthin is known to aggregate at very low concentrations and as well, astaxanthin has large third-order nonlinear optical properties [26]. Previous transmission electron microscopy studies of red aplanospores have shown that chloroplasts located in the middle and at the periphery of the cells take on a net-like appearance due to the stress-related degradation of the thylakoid membranes [13].

Previous studies have reported high concentrations of astaxanthin at the edge of aplanospores of *H. pluvialis* where astaxanthin is deposited into extra-plastidial oil bodies [4], [11]–[13]. Further, it has been speculated from measurements of Raman spectra that astaxanthin molecules form H-aggregates in the red aplanospores [15]. In order to determine the structure of astaxanthin within red aplanospores of *H. pluvialis*, polarization-dependent harmonic generation was employed.

### Structural analysis of astaxanthin within *Haematococcus pluvialis*

The organizational structure of astaxanthin in red aplanospores of *Haematococcus pluvialis* was studied using PIPO SHG and THG microscopy. Although β-carotene and astaxanthin are both present in red aplanospores and both carotenoids have large third-order nonlinear optical properties [26], previous research has indicated that significantly higher concentrations of astaxanthin are present in red aplanospores than β-carotene [6]. Therefore, it is reasonable to assume that the THG signal is generated predominantly from astaxanthin.

**Figure 3:**
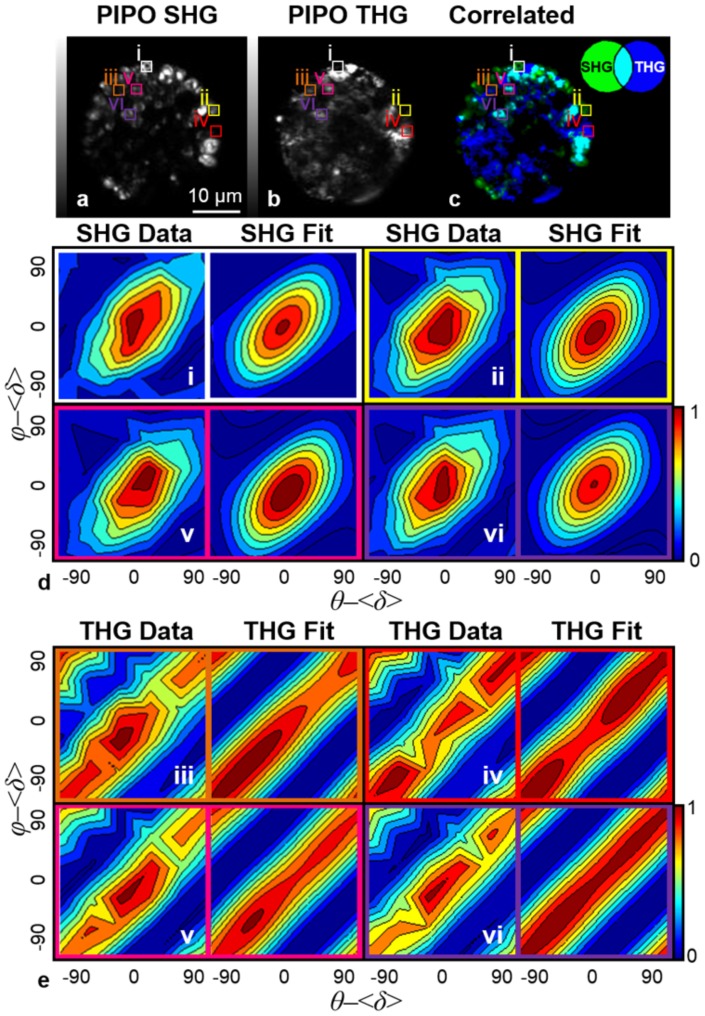
PIPO SHG and PIPO THG data of an aplanospore. An aplanospore visualized by (a) SHG, (b) THG, and (c) structural image cross-correlation analysis between SHG and THG [16]. The images consist of a summation of images recorded at various polarizations. Specific regions seen in (a, b, c) labeled as (i, ii, iii, iv, v, vi) were analyzed to produce (d, e) PIPO data contour plots and fits. Fitting of (a) PIPO SHG data for regions (i, ii, iii, iv, v, vi) were performed with equation (1) while fitting of (b) PIPO THG data for regions (i, ii, iii, iv, v, vi) were performed with equation (2). The contour plots shown of regions i and ii represent areas of intense SHG signal and no THG signal, regions iii and iv represent areas of intense THG signal and no SHG signal while regions v and vi represent areas of correlated SHG and THG signals.

PIPO measurements were performed on red aplanospores. PIPO SHG and PIPO THG data are summarized by contour plots of selected pixels shown in Fig. 3. From PIPO SHG and PIPO THG data of a red aplanospore, 6 regions were analyzed including areas where only SHG signal is produced (Fig. 3 regions i, ii), only THG signal is produced (Fig. 3 regions iii, iv), and where SHG and THG signals are correlated (Fig. 3 regions v, vi). The fitted parameters are presented in Table 1.

**Table 1:** PIPO SHG and PIPO THG fitting parameters of a red aplanospore of *Haematococcus pluvialis*.

Region	Harmonic Signal	*R_SHG_*	*R_THG1_* a	*R_THG2_*
i	SHG	3.7±0.2	-	-
ii	SHG	3.9±0.3	-	-
iii	THG	-	0.34±0.01	1.0±0.1
iv	THG	-	0.36±0.03	1.2±0.2
v	SHG and THG	3.9±0.3	0.36±0.03	1.1±0.1
vi	SHG and THG	3.7±0.3	0.35±0.02	1.0±0.1

The fitting parameters pertaining to specific regions highlighted in Fig. 3 of a red aplanospore were found. *R* ^2^ values for SHG fits were at least 0.94 and *R* ^2^ values for THG fits were at least 0.92.

a *R_THG1_* was fixed between -2 and 2, and *R_THG2_* was set as a free fitting parameter.

Fitting of the PIPO SHG data from regions i, ii, v, and vi in Fig. 3 was performed with equation (1). The *R_SHG_* ratio in all four regions was found to have an average value of 3.8±0.1. These ratio values are consistent with *R_SHG_* ratios measured for starch [39]. Since the *R_SHG_* ratios are consistent in all regions studied in Fig. 3 and are similar to the literature values, the SHG signal was confirmed to be attributed to starch present inside the algae.

Fitting of PIPO THG data from regions iii, iv, v, and vi in Fig. 3 was performed with equation (2). The values of *R_THG1_* were found to be small. Since the two ratios, *R_THG1_* and *R_THG2_*, depend on one another, the value of *R_THG1_* was fixed between −2 and 2 while *R_THG2_* was set as a free fitting parameter.

*R_THG2_* in all four regions was found to have an average value of 1.1±0.1 while *R_THG1_* was found to have an average value of 0.35±0.01. *R_THG2_* for both regions is approximately 1 while *R_THG1_* for both regions is approximately 1/3. These ratios are characteristic of the symmetry properties for an isotropic material where 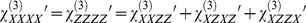 and therefore, *R_THG1_* is equal to 1/3 while *R_THG2_* is equal to 1 [40]. Thus, it is likely that astaxanthin molecules or small aggregates below the resolution of the instrument (500 nm) are generally randomly distributed in oil bodies. Areas where SHG and THG signals correlate (Fig. 3 regions v, vi) are likely regions in which astaxanthin molecules or astaxanthin aggregates surround starch granules.

### Structural analysis of H-aggregates of astaxanthin

As mentioned, it has been hypothesized that astaxanthin within red aplanospores form H-aggregates. In order to determine if the structure of astaxanthin within H-aggregates is similar to the structure of astaxanthin found in red aplanospores, PIPO THG was performed on astaxanthin H-aggregates.

**Figure 4:**
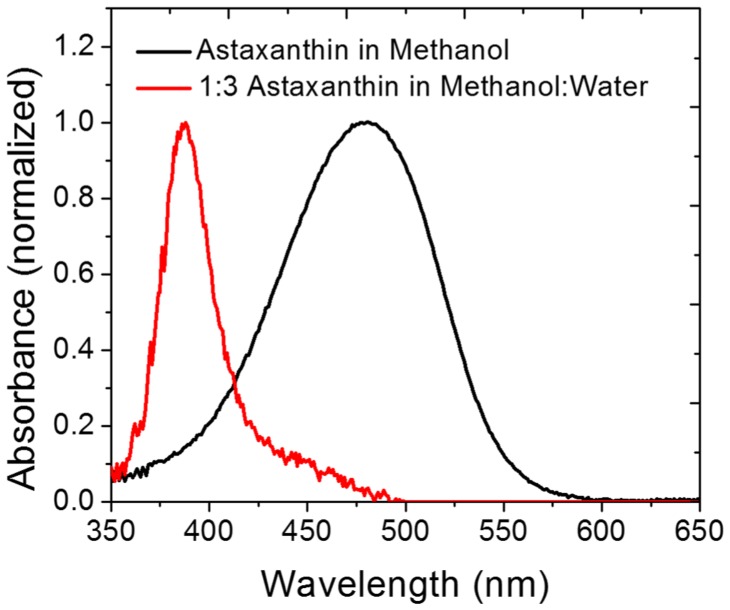
Normalized absorbance spectra of astaxanthin and astaxanthin H-aggregates. The UV-Vis spectra of astaxanthin dissolved in methanol and astaxanthin dissolved in methanol in the presence of distilled water in a ratio of 1∶3 was recorded. The absorbance peak of astaxanthin dissolved in methanol and mixed with water resulted in a blue spectral shift which is indicative of the formation of H-aggregates [14], [35].

H-aggregates of astaxanthin were formed by mixing a 50 µM solution of astaxanthin dissolved in methanol with distilled water at a ratio of 1∶3. The absorption spectrum of the mixture was taken as seen in Fig. 4. A clear indication of H-aggregates is the hypsochromic shift seen in the absorption spectrum [14], [35].

**Figure 5:**
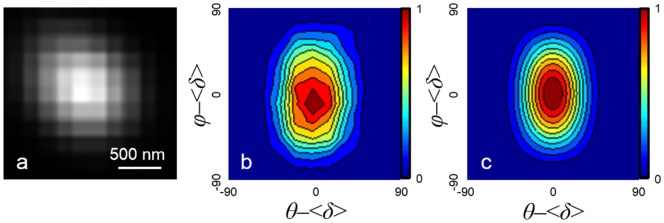
PIPO THG data of an H-aggregate of astaxanthin. (a) THG image of an astaxanthin H-aggregate along with (b) the measured PIPO THG data and (c) the PIPO THG fit performed with equation (2).

The H-aggregates were imaged with THG microscopy and the PIPO THG data of an H-aggregate was taken as exemplified in Fig. 5. The PIPO THG contour plot for a typical astaxanthin H-aggregate (Fig. 5b) resembles an upright oval and is significantly different than the PIPO THG contour plot for astaxanthin contained in oil bodies of *H. pluvialis* (Fig. 3e regions iii, iv, v, vi) which resembles diagonal lines. Therefore, the structure of astaxanthin within the synthetic H-aggregate is different than the structure of astaxanthin within oil bodies of *H. pluvialis*. This is further evidenced by the changes in the third-order nonlinear optical susceptibility ratios where *R_THG1_* is equal to 1.0±0.4 and *R_THG2_* is equal to 16±5 (*R* ^2^ value of 0.964) for the *in vitro* astaxanthin H-aggregate. Since *R_THG2_* is much larger than *R_THG1_*, this means that the 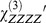 component is large, thus indicating that the polarization of the incident laser along the Z′-axis results mostly in radiation along the Z′-axis. Therefore, the alignment of astaxanthin molecules in the H-aggregate is uniaxial where it is presumed that the dominating molecular nonlinear dipole for astaxanthin is along the conjugated backbone.

The PIPO THG contour plot for the astaxanthin H-aggregate in Fig. 5 b, c is similar to the PIPO THG contour plots of J-aggregates (data not shown), and β-carotene crystalline aggregates found in orange carrot [28]. Therefore, the alignment of astaxanthin molecules in both H- and J-aggregates is uniaxial.

The comparative analysis of PIPO THG data between *in vivo* and *in vitro* astaxanthin structures shows that astaxanthin within oil bodies of *Haematococcus pluvialis* (Fig. 3e regions iii, iv, v, vi) is different from the *in vitro* aggregates (Fig. 5 b, c) in that the *in vivo sample* shows no long-range order. The elongated PIPO plot (Fig. 3e regions iii, iv, v, vi) can occur due to the isotropic arrangement of astaxanthin molecules if they are dissolved in oil droplets or if small crystalline aggregates (<∼100 nm) are distributed isotropically within the oil bodies. Overall, this study provides further evidence that PIPO SHG and THG can be used to obtain information on the structural organization of biological aggregates *in vivo*.

## Conclusions

The localization of astaxanthin accumulation in *Haematococcus pluvialis* can be readily studied with multicontrast nonlinear optical microscopy. Under stress conditions, astaxanthin accumulates in the central region of aplanospores. Polarization-dependent THG studies of red aplanospores reveal that astaxanthin is isotropically arranged in oil bodies. In contrast, synthesized H- and J-aggregates of astaxanthin demonstrate uniaxial alignment of astaxanthin molecules. Since carotenoids tend to form H- and J-aggregates readily in aqueous solutions, this research implies that astaxanthin present in biological organisms undergoes a distinctly different way of packing likely due to the physical environment of the alga or is controlled enzymatically. PIPO SHG and THG microscopy allows convenient *in vivo* measurements of the dynamics of astaxanthin accumulation in algal species, and when subjected to different conditions can be used to select algae to maximize the extraction yield of carotenoids for aquaculture, nutraceutical and pharmaceutical industries.
