# Correction: Organization of Astaxanthin within Oil Bodies of *Haematococcus pluvialis* Studied with Polarization-Dependent Harmonic Generation Microscopy

**DOI:** 10.1371/journal.pone.0121773

**Published:** 2015-03-31

**Authors:** The PLOS ONE Staff

In the “Theoretical Methods” section of the Materials and Methods, there is an error in the second equation. Please view the complete, correct equation here:

$$I_{3\omega }\propto |\sin\varphi '\sin\theta '\left(\cos^{2}\theta '\left(3R_{THG1}-1\right)+1\right)+{\cos\varphi '\cos\theta '\left(3R_{THG1}-\cos^{2}\theta '\left(3R_{THG1}-R_{THG2}\right)\right)|}^{2}$$ (2)
